# A compact metasurface-based circularly polarized antenna with high gain and high front-to-back ratio for RFID readers

**DOI:** 10.1371/journal.pone.0288334

**Published:** 2023-08-22

**Authors:** Hung Tran-Huy, Huy-Hoang Nguyen, Thao Hoang Thi Phuong

**Affiliations:** 1 Faculty of Electrical and Electronic Engineering, PHENIKAA University, Hanoi, Vietnam; 2 PHENIKAA Research and Technology Institute (PRATI), Hanoi, Vietnam; 3 Faculty of Electronics and Telecommunications, Electric Power University, Hanoi, Vietnam; Edinburgh Napier University, UNITED KINGDOM

## Abstract

Developing a compact circularly polarized (CP) antenna with good radiation characteristics for handheld radio frequency identification (RFID) readers is a very challenging task. Many compact CP antennas have been reported in the open literature, but most suffer from critical drawbacks of low gain and/or high back radiation. This paper presents a metasurface (MS) based CP antenna with compact size, high gain, and high front-to-back ratio characteristics. The compact size of the proposed design is achieved by using a 2 × 2 unit-cell MS, while the CP realization is accomplished through a coupling between the MS and a Y-shaped patch as a primary CP source. The final antenna has compact overall dimensions of 0.45λ × 0.45λ × 0.02λ, where λ is the guided wavelength at the center frequency. The operating bandwidth is about 2.0% (2.43–2.48 GHz) and the broadside gain is about 6.3 dBi. Besides, the front-to-back ratio (FBR) defined by the difference gain levels between the forward and backward directions is about 18 dB. Compared with the related compact CP antennas in the literature, the proposed design has the advantages of high gain and high FBR, making it suitable for compact RFID readers.

## Introduction

Radio Frequency Identification (RFID) is a wireless communication system consisting of two components: tags and readers [1]. This kind of technology, which uses electromagnetic waves to exchange information between the tags and the readers, has been widely used in tracking systems. The tag antennas are normally linearly polarized (LP) and randomly positioned. Thus, the reader antennas are usually designed with circularly polarized (CP) operation to avoid polarization mismatch [2]. Numerous CP antennas have been proposed for modern compact and lightweight RFID readers. However, compact size will lead to low gain radiation. Besides, the increase in back radiation or low front-to-back ratio (FBR) also decreases the anti-multipath interference ability of the CP antenna. Therefore, designing a CP antenna with a compact size, high gain, and low back radiation is challenging.

Theoretically, CP radiation can be achieved when the antenna is excited by two orthogonal electric fields with equal amplitude and phase difference of 90°. In [3–6], multi-fed methods, such as 3-dB orthogonal branch coupler [3, 4], Wilkinson power divider [5], and 180° power splitter [6], can easily achieve circular polarization. They required large spaces and complicated feeding networks, which are not appealing to compact handheld RFID readers. Less complexity is achieved when using the single-fed scheme [7–9]. However, large overall dimensions are still the critical disadvantage of these antennas.

Various techniques have been developed to minimize the CP antenna for RFID applications. In [10], a single-fed dual-layer planar inverted-F antenna (PIFA) is proposed. Although compact dimensions are obtained, this design also suffers from low gain and low FBR. For better performance, another miniaturization method is by etching slots in the radiating square-shaped patch [4, 11]. Alternatively, fractal geometries are also presented for size reduction [12, 13]. Recently, metamaterial resonator-type and metamaterial-inspired antennas have received substantial research interest for miniaturization purposes. The vertical split-ring resonator (SRR) and complementary split-ring resonator (CSRR) are proposed in [14, 15]. In [16], a patch antenna is loaded with the composite right/left-handed (CRLH) mushroom-like structures and a reactive impedance surface (RIS). Apart from the abovementioned methods, antenna miniaturization can also be obtained by using metal-insulator-metal (MIM) capacitors [17] or high permittivity substrate [18]. In general, these designs have the advantage of compact size. However, there are several existent drawbacks including high profile, low gain, and low FBR.

The motivation of this paper is to design a compact directional CP antenna with high gain and high FBR. To achieve this, the antenna realization is as follows:

- To achieve compact and directional beam antenna, microstrip patch and MS-based antenna are considered.
- To achieve high gain, the MS-based antenna is a better option than the patch antenna. This is due to the large radiating aperture of the MS in comparison with the patch.
- To achieve high FBR or small back radiation, the MS-based antenna has more advantages than the patch antenna. For the patch, the back radiation is caused by the diffraction of the surface wave at the edges of the ground plane. Smaller ground plane results in higher back radiation. In contrast, the electromagnetic waves are confined between the MS layer and the ground plane layer for the MS-based antenna. This contributes to reducing back radiation.

Accordingly, the proposed antenna is based on a 2 × 2 unit-cell metasurface (MS) and fed by a Y-shaped patch as a CP source. To demonstrate the feasibility of the proposed concept, measurements are implemented on a fabricated antenna prototype. The measured data show an operating bandwidth (BW) of 2.0% (2.43–2.48 GHz). The antenna with a compact size of 0.45λ x 0.45λ x 0.02λ can achieve a high gain radiation of 6.4 dBi and a high FBR of 18 dB.

## Metasurface design

**Fig 1:**
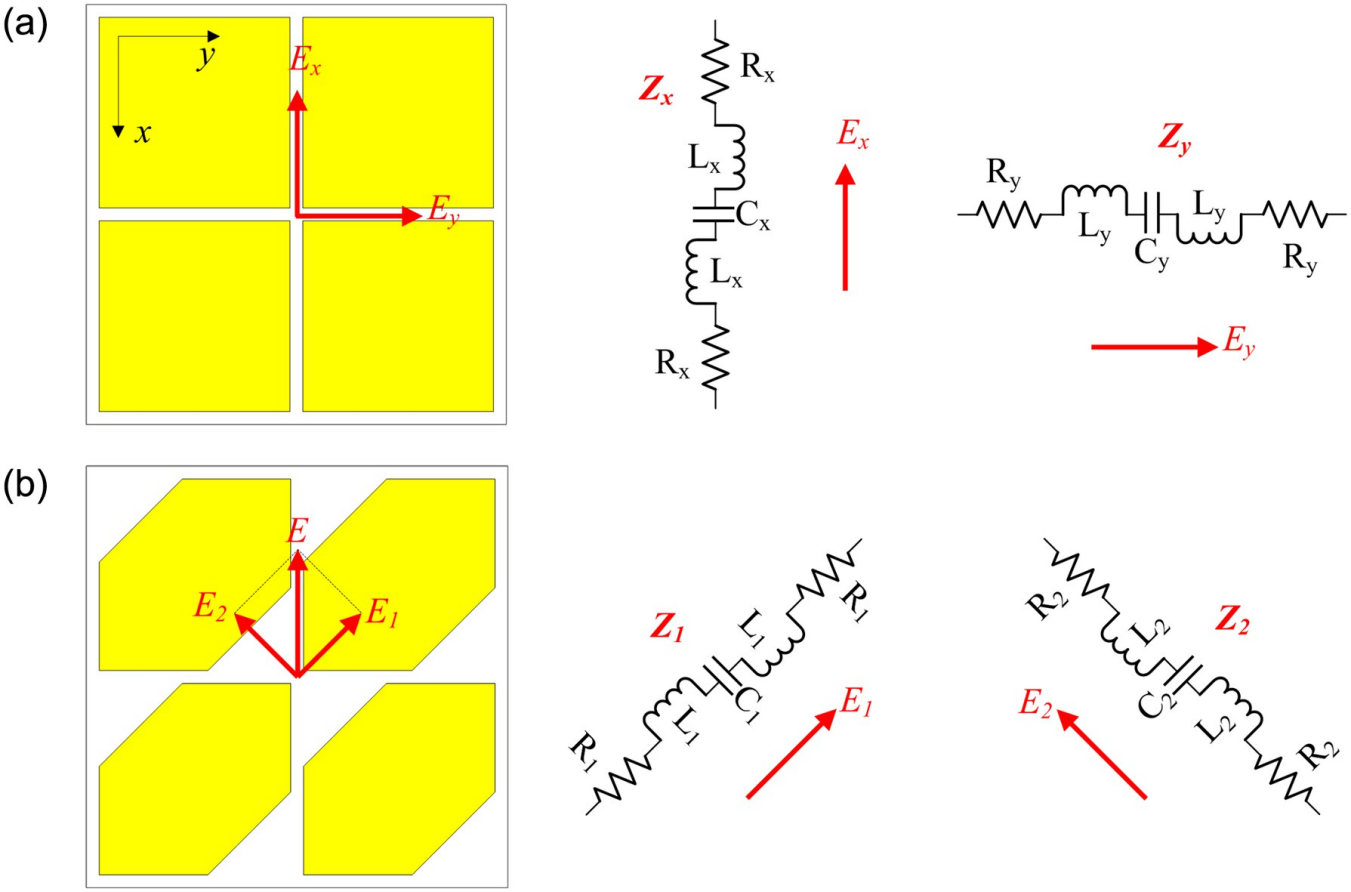
Equivalent circuit of different MS configurations. (a) Symmetric unit-cell MS, (b) asymmetric unit-cell MS.

The MS-based antenna is one kind of MS application that employed the resonant modes of finite MS. Various MS antennas have been developed for wideband, low profile, or circular polarization applications [19–24]. In the MS-based antenna, the MS is the primary radiating aperture of the antenna. The antenna’s operation characteristics are strongly dependent on this surface. There are two ways to produce CP radiation for the MS-based antenna, as shown in Fig 1. In the first case, the unit cell has a symmetric structure. The equivalent circuits in the *x*- and *y*-direction are identical, *Z*_*x*_ = *Z*_*y*_. Thus, the CP operation can be achieved when a CP source (with two orthogonal fields, *E*_*x*_ and *E*_*y*_, and 90^0^ out of phase) is used to excite this kind of MS. In the second case, the unit cell has an asymmetric structure. To have CP operation, this kind of MS is commonly excited by a linear source, for example, the vertical polarization in Fig 1(b). The MS layer acts as a polarization conversion to transform the LP source into the CP wave. Note that the unit cell in this case has various shapes and the shape in Fig 1(b) is just one example. Here, the E-field from the LP source is broken into two orthogonal fields, *E*_1_ and *E*_2_. By controlling the truncated corners, the impedances *Z*_1_ and *Z*_2_ can be equal in magnitude and 90^0^ out of phase. Consequently, CP realization can be accomplished.

In this paper, a symmetric MS is utilized. The characteristic mode analysis (CMA) is utilized to predict the modal behaviors and operation characteristics of the MS [25, 26]. To have efficient CP radiation in the broadside direction, the symmetric MS must support two modes and the following conditions need to be satisfied:

- They are identical in modal significance.
- The current distributions are orthogonal arrangements.
- The broadside directivities are similar.

**Fig 2:**
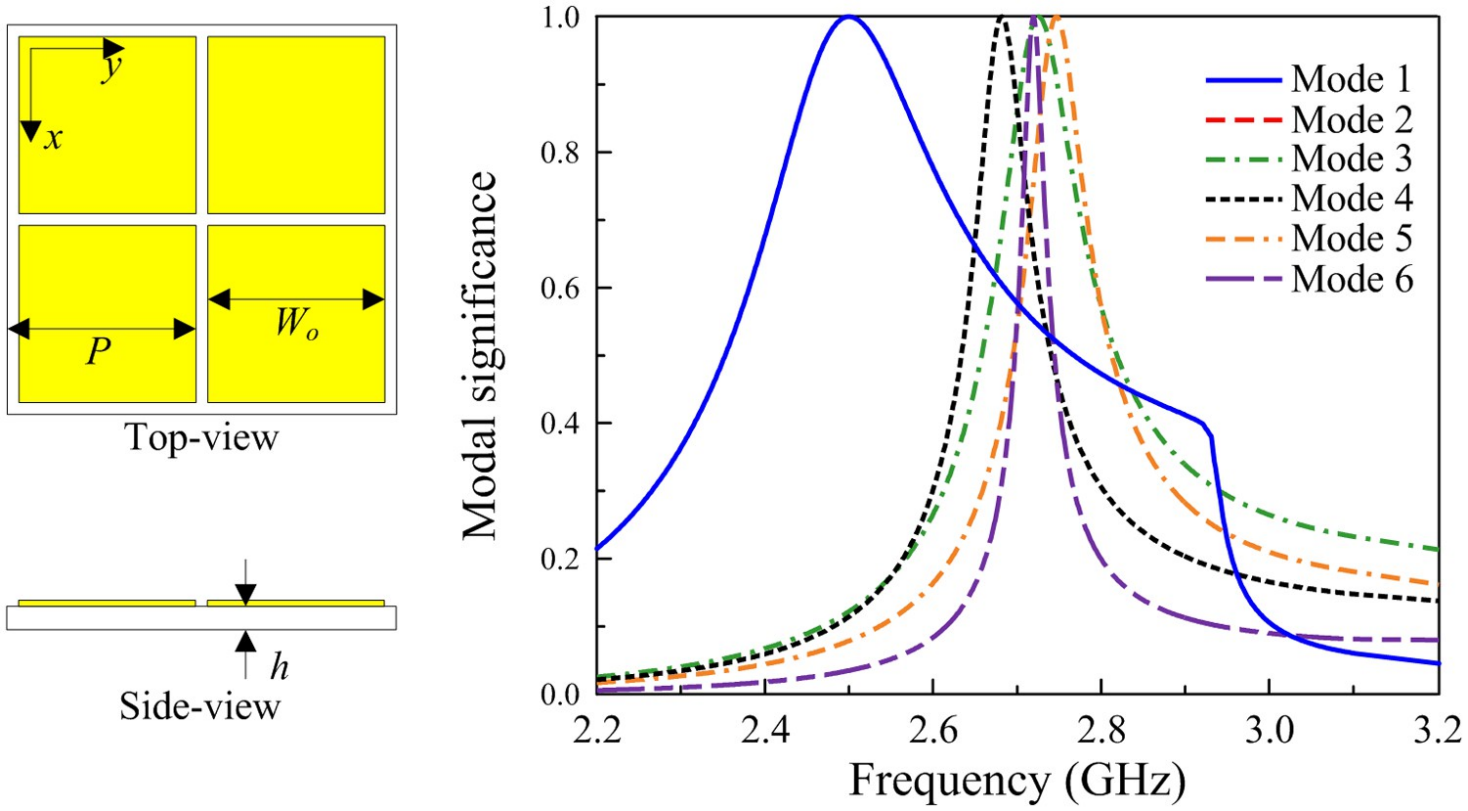
Simulated modal significance of the utilized 2 × 2 unit cell MS with *P* = 27.5 mm, *W*_0_ = 27.3 mm, and *h* = 3.04 mm.

**Fig 3:**
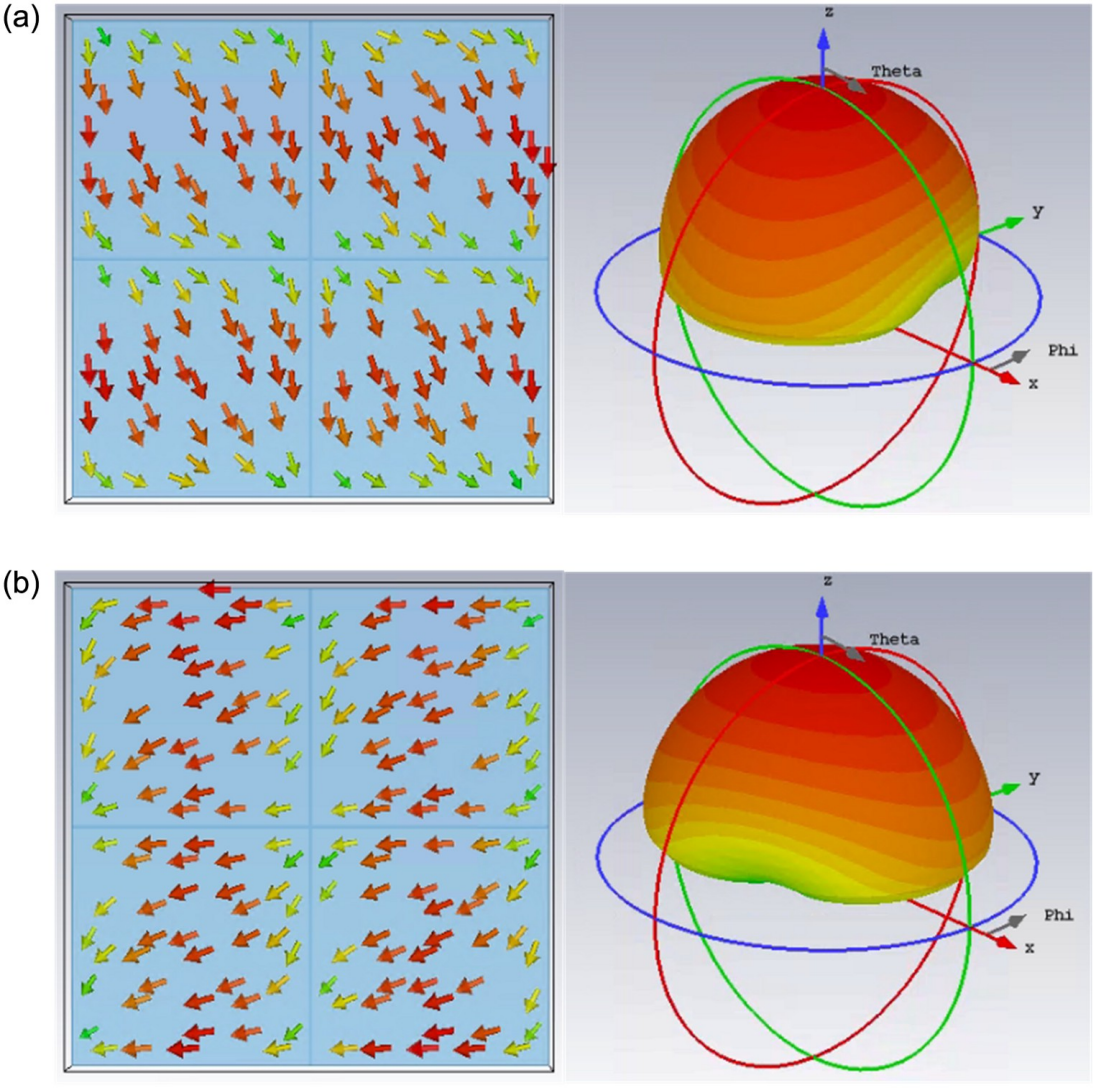
Simulated surface current and far-field of (a) Mode 1, and (b) Mode 2.

Note that many MS-based CP antennas have been reported in the literature [27–30]. However, they just focus on hows to achieve wideband and high gain operation with at least 4 × 4 unit-cell MS. Thus, these antennas suffer from critical drawbacks of a large footprint of bigger than 0.7λ × 0.7λ and the profiles of such antennas are higher than 0.05λ. In contrast, this paper doesn’t focus on wideband operation. This paper focuses on hows to achieve a compact size while having a high gain radiation and a high FBR. This distinguishes the presented work from the others. To achieve compact size, a 2 × 2 unit-cell MS is used as a radiating aperture of the proposed compact CP antenna, as presented in Fig 2. The CMA on the utilized MS is carried out using the MoM-based CMA tool in the commercial simulation software CST MWS. The modal significances of the first 6 modes from 2.0 to 3.2 GHz are calculated and sorted at 2.2 GHz, as illustrated in Fig 2. It is noted that the mode resonates and radiates the most efficiently when the modal significance is equal to 1. In the interested frequency range around 2.5 GHz, Mode 1 and Mode 2 have identical modal significances of approximately 1. Besides, Mode 1 and Mode 2 also provide good broadside direction, and the current distributions are in perpendicular arrangement, as illustrated in Fig 3. Therefore, it can be concluded that the utilized 2 × 2 MS has the potential to produce CP radiation around 2.5 GHz. Note that the other modes (Mode-3, -4, -5, -6) have high modal significances around 2.7 GHz and don’t have broadside radiation patterns at this frequency. Therefore, these modes are not utilized.

## Compact CP MS-based antenna design

**Fig 4:**
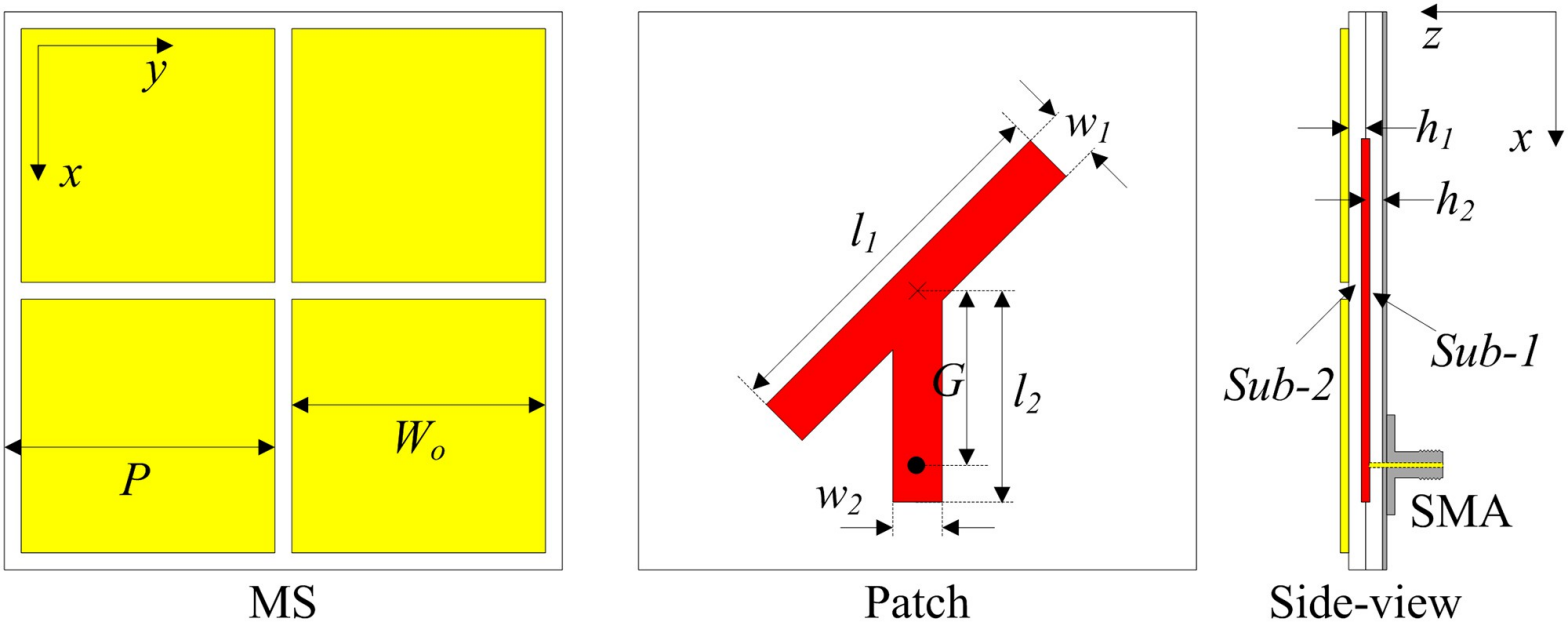
Geometry of the proposed compact MS-based CP antenna. *P* = 27, *W*_0_ = 27.1, *h*_1_ = *h*_2_ = 1.52, *l*_1_ = 37, *w*_1_ = 5, *l*_2_ = 21, *w*_2_ = 5, *G* = 20 (unit: mm).

The configuration of the proposed compact CP antenna is presented in Fig 4. The antenna consists of three layers including a ground, a CP source, and an MS. These layers are designed on two 1.52-mm-thick Taconic RF-35 substrates with a dielectric constant of 3.5. The ground plane and the CP radiating source are respectively printed at the bottom and top sides of Sub-1. Meanwhile, the MS layer consists of 2 × 2 unit cells is printed at the top side of Sub-2. As inspiration from [31], the CP source is a Y-shaped patch. Note that the CP source can be the conventional CP structures such as truncated corner square patches, diagonal slotted square/circular patches, and so on. The antenna is excited through the SMA connector, whose inner and outer conductors are respectively connected to the radiating patch and the ground plane.

**Fig 5:**
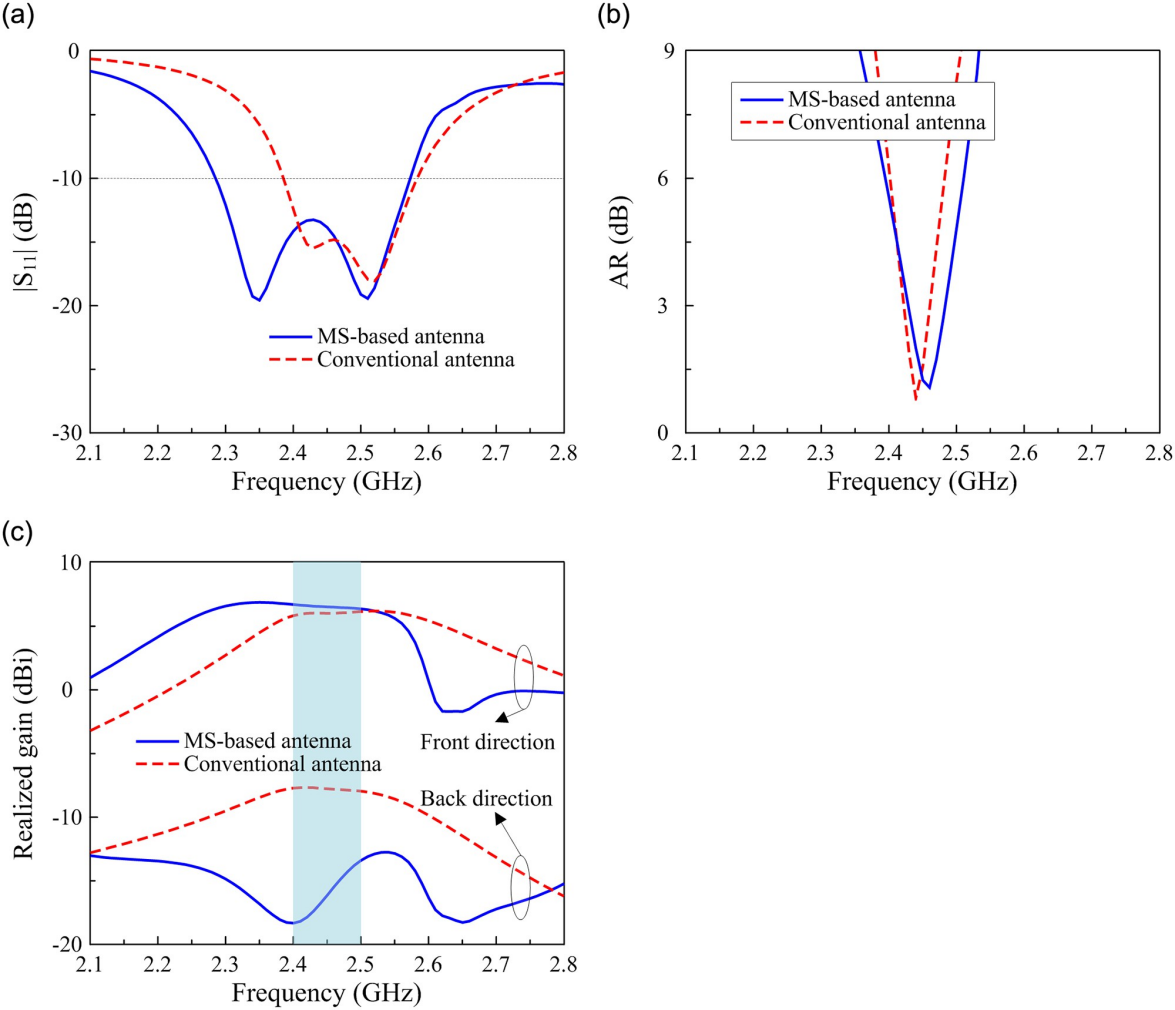
Simulated performance of different antennas. (a) |*S*_11_|, (b) AR, and (c) realized gain.

To demonstrate the effectiveness of the utilized concept, the performances of the proposed MS-based antenna and the conventional CP patch are compared. For a fair comparison, the conventional CP square patch with truncated corners is designed on a Taconic RF-35 substrate with similar overall dimensions to the MS-based antenna. Fig 5 shows the simulated reflection coefficient |*S*_11_|, AR, and realized gain of these two designs. Around 2.45 GHz, both antennas show good impedance matching with |*S*_11_| of less than –10 dB and good CP radiation with an axial ratio (AR) of lower than 3 dB. However, the antenna gain observes a strong difference. For the conventional CP patch antenna, surface wave diffraction at the edges of the ground plane increases the back radiation. For the proposed structure, the electromagnetic waves radiated by the MS at its resonant frequency are confined into the MS through the magnetic coupling effect. When the MS is illuminated by the primary source, the radiated field is spread over a larger radiating aperture than the conventional design. Therefore, the antenna’s gain in the forward direction increases. The back radiation is also further suppressed, leading to high FBR.

**Fig 6:**
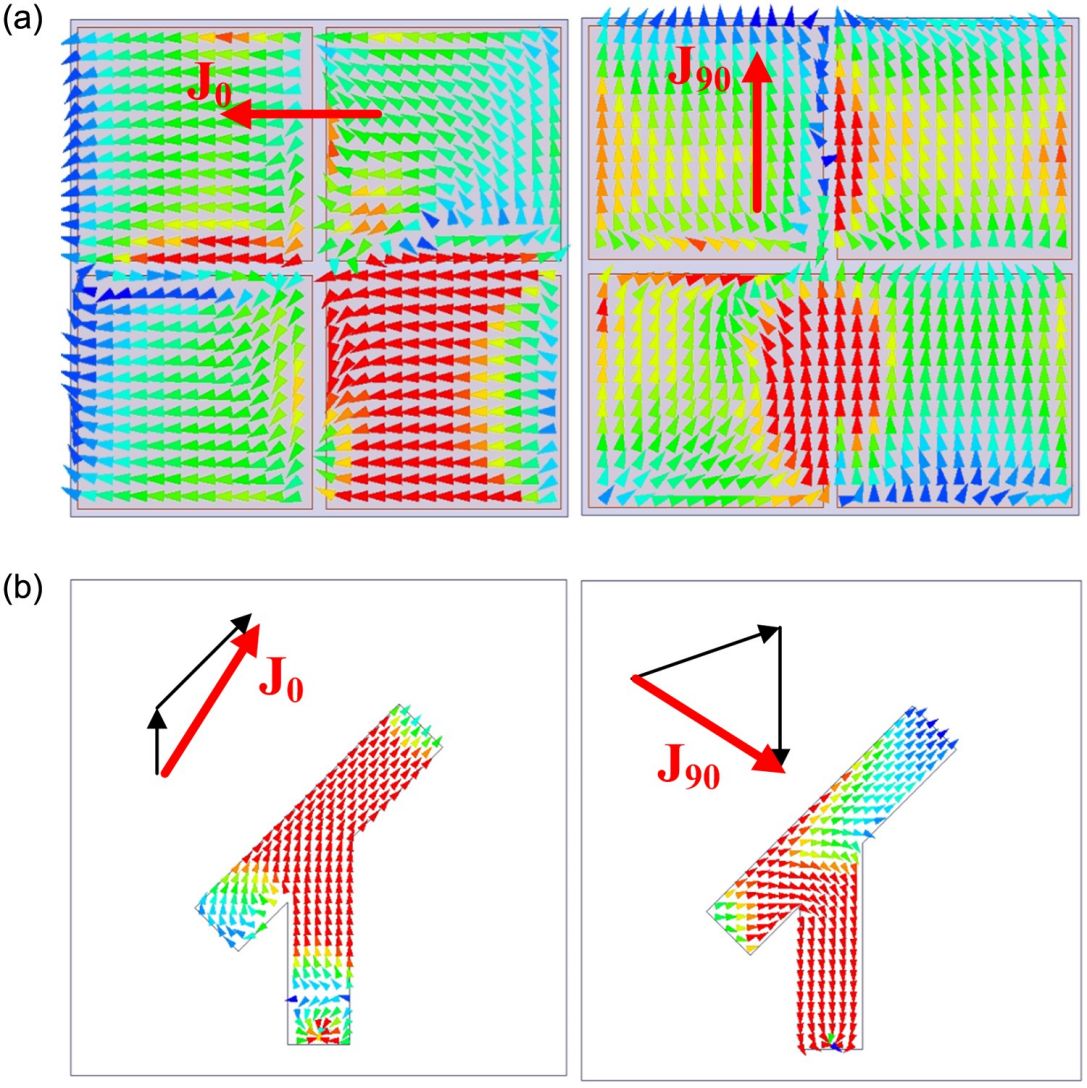
Simulated current distributions at 2.45 GHz. (a) MS, (b) CP source.

The CP realization of the proposed antenna can be verified based on the simulated current distributions on the CP source and the MS at 2.45 GHz. Fig 6 shows the simulated current distributions at different phases. In terms of the CP source, when the phase changes from 0° to 90°, the vector currents are orthogonal, and the rotation is clockwise. Similarly, two orthogonal fields are also observed in the MS. The rotation of vector current is clockwise, which is evident in the left-hand CP (LHCP) realization.

## Antenna optimization

### Operating band

**Fig 7:**
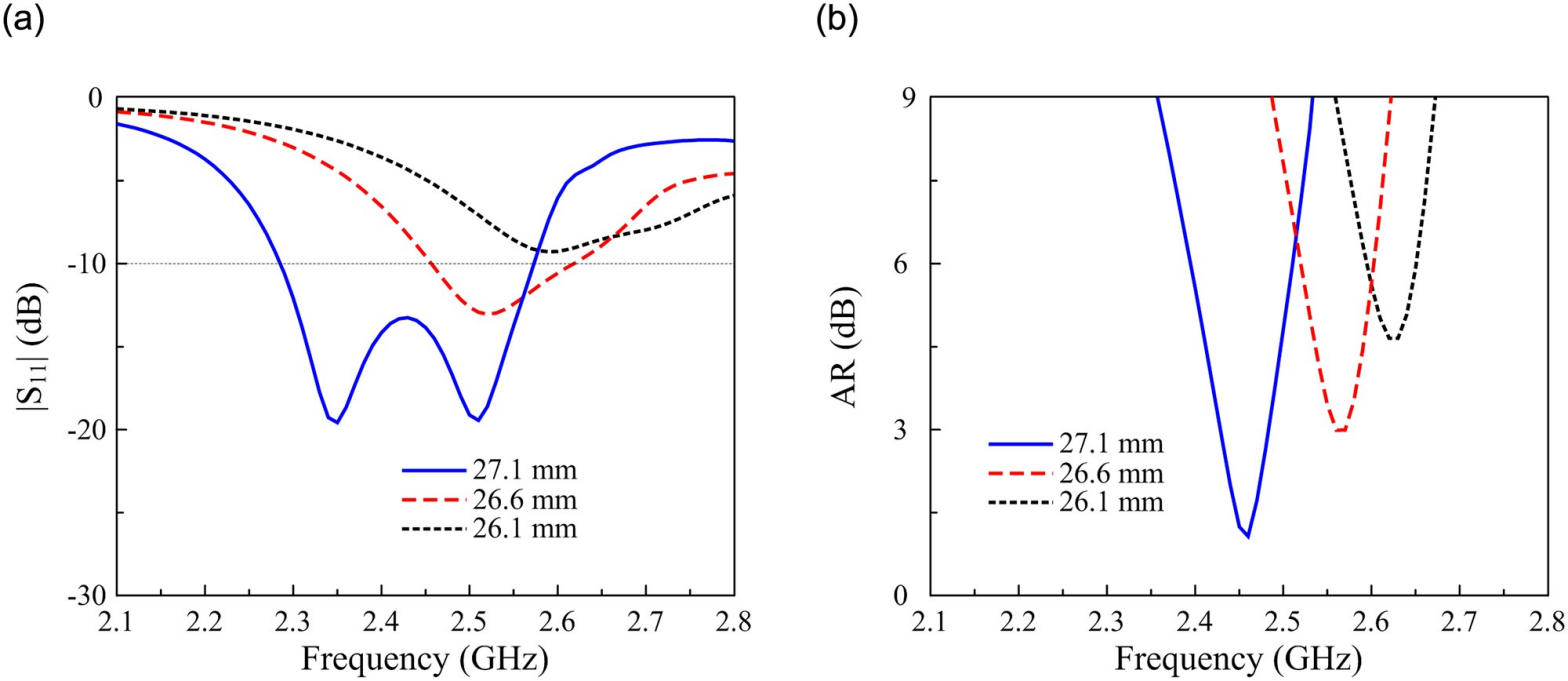
Simulated (a) |*S*_11_| and (b) AR of the proposed antenna for different values of *W*_0_.

Since the MS is the primary radiating aperture of the proposed antenna, the operating band is theoretically determined by the size of the unit cell, *W*_0_. Fig 7 presents the simulated |*S*_11_| and AR characteristics for different values of *W*_0_. The data indicate that changing *W*_0_ significantly affects the operating frequency band. Both impedance matching and AR bands shift upwards when decreasing *W*_0_.

### Axial ratio

**Fig 8:**
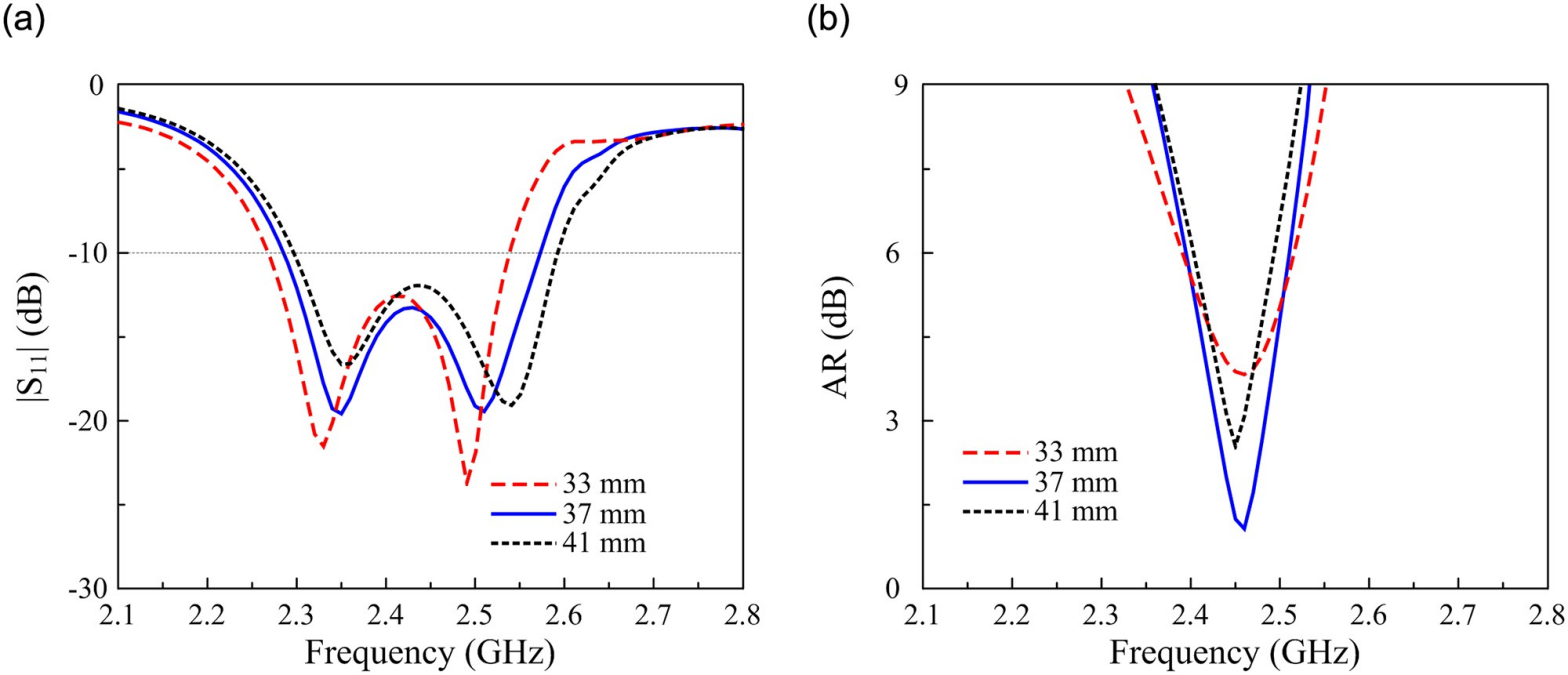
Simulated (a) |*S*_11_| and (b) AR of the proposed antenna for different values of *l*_1_.

Due to the symmetrical MS, the CP radiation of the proposed antenna is realized by the CP source Y-shaped patch. Thus, the CP operation is optimized through the dimensions of this patch. Fig 8 shows the simulated |*S*_11_| and AR results against the variation of the patch’s length, *l*_1_. As observed, the AR is significantly affected by *l*_1_. Meanwhile, the impedance performance is almost stable with the variation of *l*_1_.

### Impedance matching

**Fig 9:**
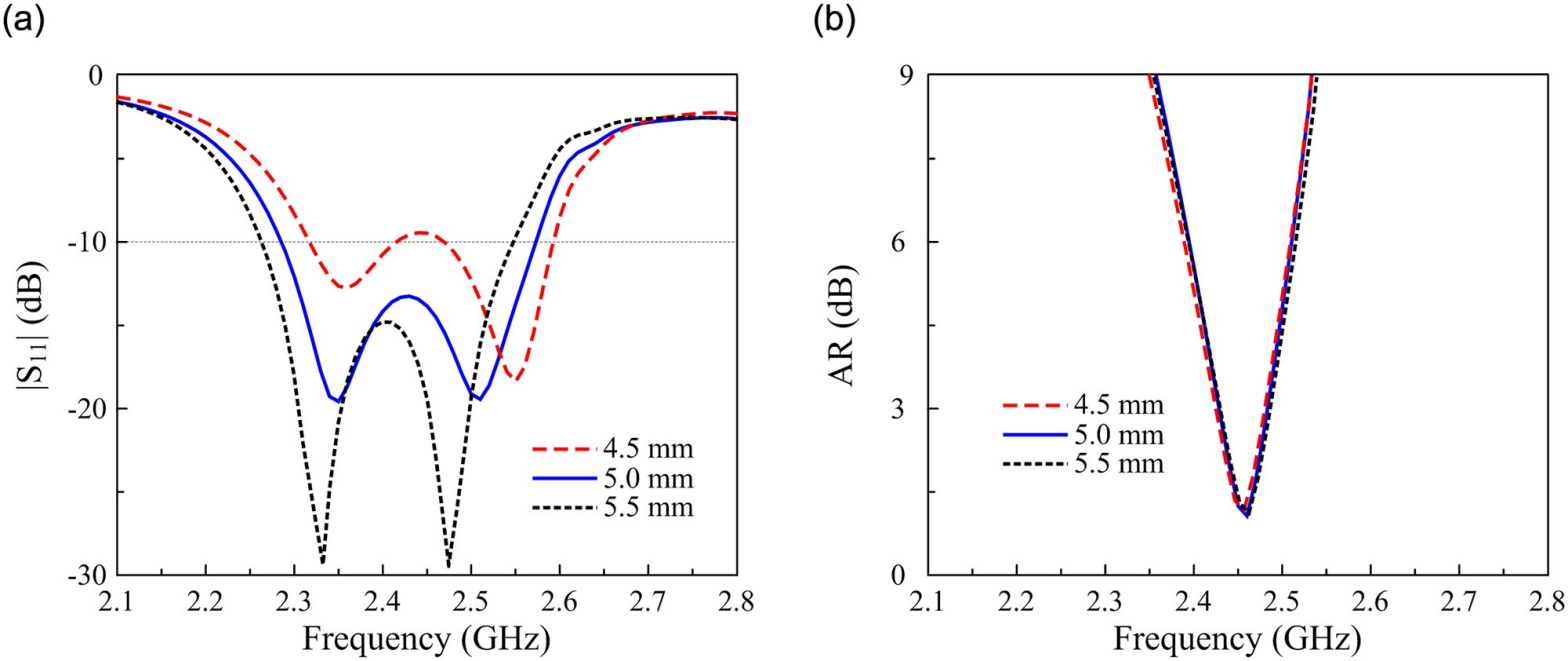
Simulated (a) |*S*_11_| and (b) AR of the proposed antenna for different values of *w*_2_.

**Fig 10:**
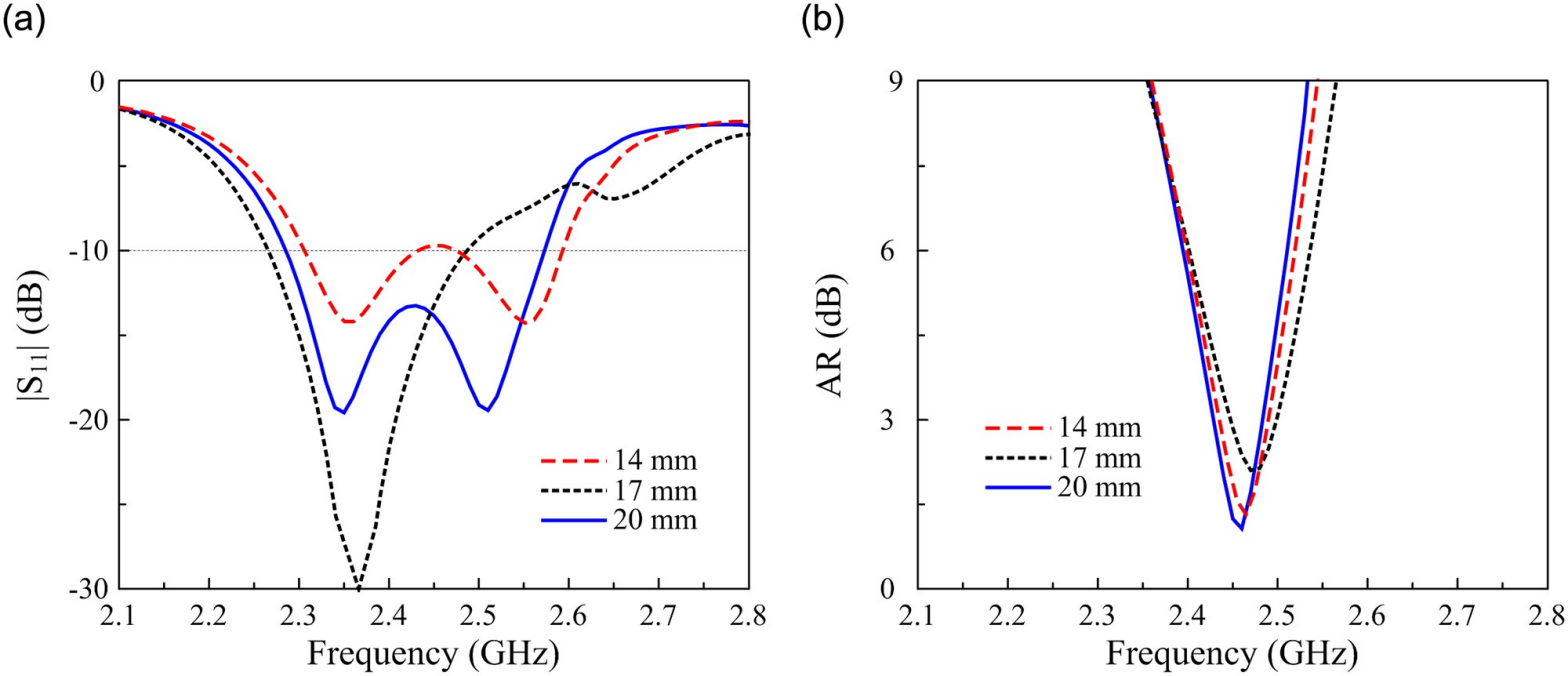
Simulated (a) |*S*_11_| and (b) AR of the proposed antenna for different values of *G*.

The impedance matching is strongly determined by the width, *w*_2_, and the feeding position, *G*. The effect of *w*_2_ and *G* on |*S*_11_| and AR performance is presented in Figs 9 and 10. It can be seen obviously that both *w*_2_ and *G* have a minor effect on AR performance. The 3-dB AR BW remains unchanged with different values of these parameters. On the other hand, the impact on the matching performance is remarkable. The antenna shows good impedance matching with proper values of *w*_2_ and *G*.

## Measurement results

To demonstrate the effectiveness and feasibility of the proposed antenna, a prototype of the antenna was fabricated and measured. The reflection coefficient is measured by a Vector Network Analyzer and the radiation features are implemented in the far-field microwave anechoic chamber.

**Fig 11:**
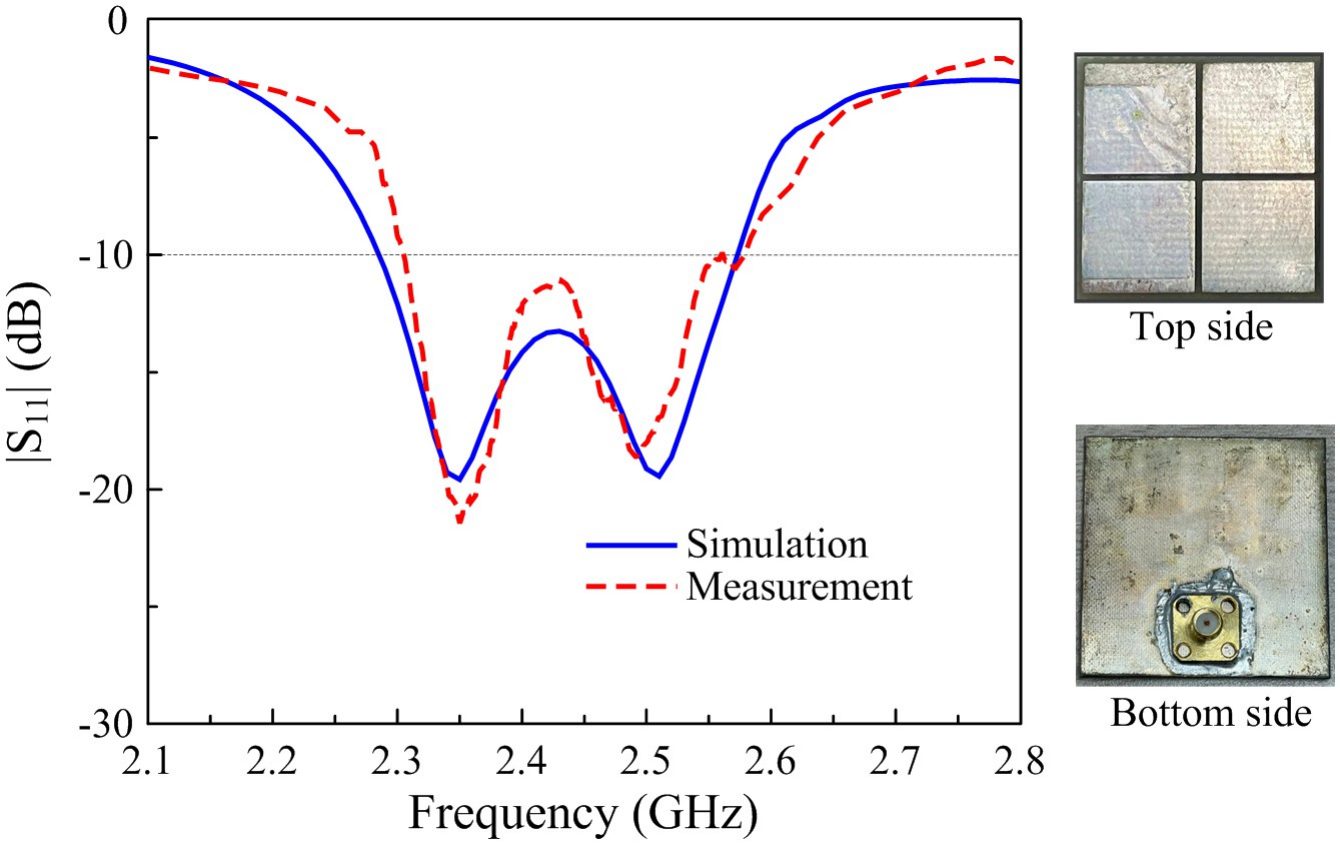
Simulated and measured |*S*_11_| of the proposed antenna.

**Fig 12:**
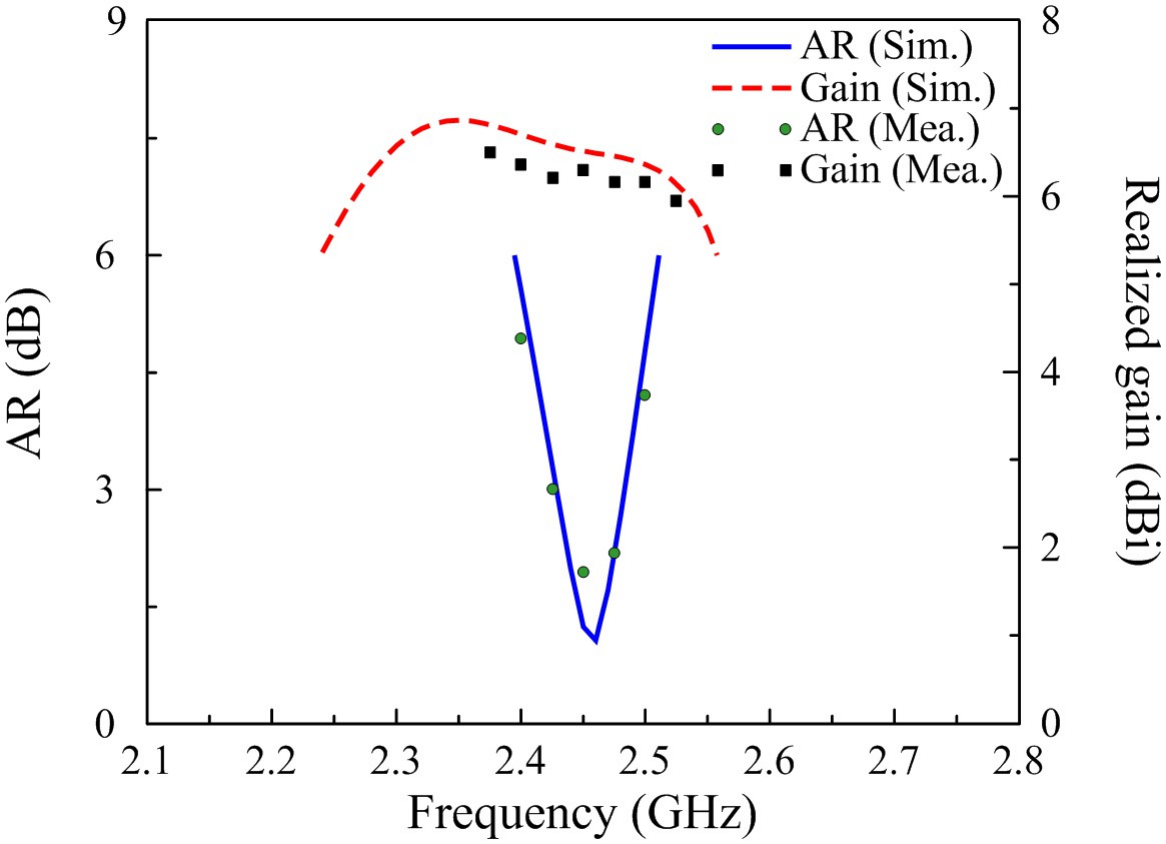
Simulated and measured AR and gain of the proposed antenna.

Fig 11 illustrates the simulated and measured reflection coefficients of the proposed antenna. The measured result shows that the BW for |*S*_11_| smaller than -10 dB covers a frequency band from 2.30 to 2.57 GHz. Fig 12 presents the simulated and measured ARs and realized gains in the broadside direction of the proposed antenna. The measured AR of less than 3 dB is from 2.43 to 2.48 GHz, which is fully covered by the impedance BW. The gain across this band is about 6.3 dBi. Some discrepancies between simulation and measurement results are mainly caused by the tolerances in fabrication and unideal measurement conditions. Additionally, the antenna also has simulated radiation efficiency of better than 85% over the operating frequency band.

**Fig 13:**
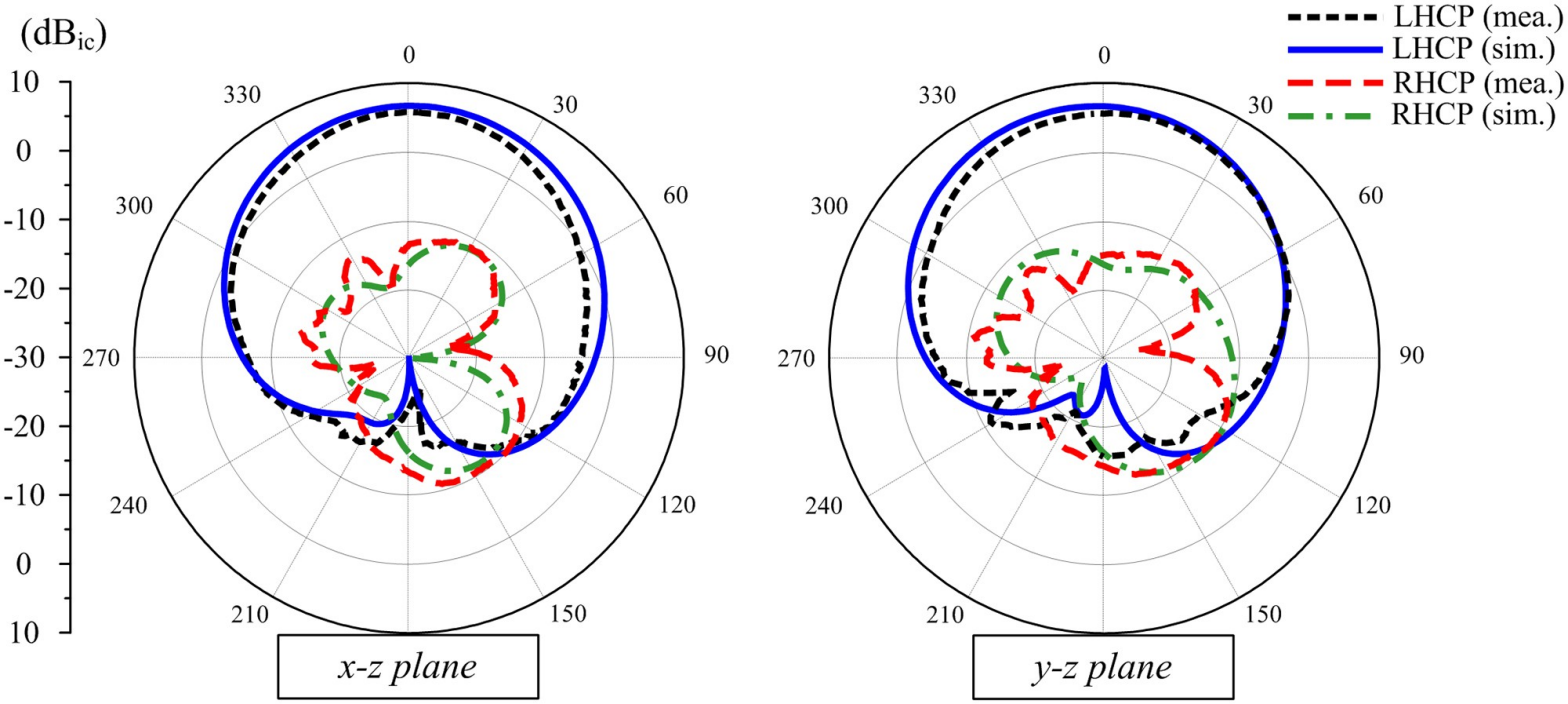
Simulated and measured radiation patterns at 2.45 GHz of the proposed antenna.

Fig 13 shows the simulated and measured radiation patterns at 2.45 GHz in both principal planes of *x* − *z* and *y* − *z*. The measured results show that the proposed antenna has a good radiation pattern, which is quite symmetric around the broadside direction. The FBR is better than 18 dB and the cross-polarization is 20 dB less than the co-polarization.

Finally, a comparison of the proposed design with several representative published studies is summarized and tabulated in Table 1. Note that the antenna size is defined in terms of wavelength at the center operating frequency of the whole design, including both radiating element and ground plane. The proposed MS-based CP antenna could acquire a compact size as the other works. However, there are certain improvements in the proposed design including high gain and high FBR as well. Most of the reported works have a low gain of less than 6.0 dBi and the FBR is generally less than 10 dB. Additionally, it is also worth noting that the proposed work shows the best FBR.

**Table 1:** Comparison among the compact CP antennas.

Ref.	Antenna size (λ)	Appli.	Applied method	Oper. band (GHz)	BW (%)	Max. Gain (dBi)	FBR (dB)
[10]	0.36 × 0.32 × 0.05	RFID	PIFA	0.43	1.1	3.8	8
[14]	0.45 × 0.45 × 0.06	RFID	Ring patch	0.86	24.2	5.6	14
[11]	0.36 × 0.36 × 0.05	RFID	Grounded patch	0.92	4.9	5.5	10
[12]	0.16 × 0.16 × 0.01	RFID	Fractal patch	0.91	<1.0	5.5	N/G
[13]	0.55 × 0.55 × 0.01	RFID	Fractal patch	2.45	0.2	6.9	18
[14]	0.46 × 0.46 × 0.03	RFID	Vertical SRR	0.91	1.4	4.3	7.5
[15]	0.46 × 0.46 × 0.05	RFID	Crossed SRR	0.91	0.8	5.5	8
[16]	0.30 × 0.30 × 0.03	WLAN	CRLH + RIS	2.58	1.5	3	9
[17]	0.46 × 0.46 × 0.06	RFID	MIM capacitors	0.92	0.5	4.3	10
Prop.	0.45 × 0.45 × 0.02	RFID	2 × 2 MS	2.45	2	6.4	18

## Conclusion

A compact CP antenna with high gain and high FBR for RFID readers is presented and investigated in this paper. The proposed design is based on the 2 x 2 unit-cell MS and the Y-shaped patch as the primary CP radiating source. The proposed antenna with compact dimensions of 0.45λ x 0.45λ x 0.02λ achieves an operating BW of 2.0% (2.43—2.48 GHz). A high broadside gain of 6.3 dBi and a high FBR of 18 dB are also accomplished. In comparison with other related works, the antenna has the advantages of high gain and high FBR while having similar overall dimensions. The proposed antenna can be a good candidate for compact RFID readers.
